# JAK Inhibition Prevents DNA Damage and Apoptosis in Testicular Ischemia-Reperfusion Injury via Modulation of the ATM/ATR/Chk Pathway

**DOI:** 10.3390/ijms222413390

**Published:** 2021-12-13

**Authors:** Farah Khashab, Farah Al-Saleh, Nora Al-Kandari, Fatemah Fadel, May Al-Maghrebi

**Affiliations:** Department of Biochemistry, Faculty of Medicine, Kuwait University, Safat 13110, Kuwait; dashtikh91@gmail.com (F.K.); farah.saud92@gmail.com (F.A.-S.); norayaq@gmail.com (N.A.-K.); fatma.alshaty@hotmail.com (F.F.)

**Keywords:** ischemia-reperfusion injury, apoptosis, oxidative stress, JAK/STAT, DNA damage response, ATM, ATR

## Abstract

Testicular ischemia reperfusion injury (tIRI) causes oxidative stress-induced DNA damage leading to germ cell apoptosis (GCA). The aim of the study is to establish a direct link between JAK2 activation and the DNA damage response (DDR) signaling pathways and their role in tIRI-induced GCA using AG490, a JAK2 specific inhibitor. Male Sprague Dawley rats (*n* = 36) were divided into three groups: sham, unilateral tIRI and tIRI + AG490 (40 mg/kg). During tIRI, augmentation in the phosphorylation levels of the JAK2/STAT1/STAT3 was measured by immunohistochemistry. Observed spermatogenic arrest was explained by the presence of considerable levels of DSB, AP sites and 8OHdG and activation of caspase 9, caspase 3 and PARP, which were measured by colorimetric assays and TUNEL. The ATM/Chk2/H2AX and ATR/Chk1 pathways were also activated as judged by their increased phosphorylation using Western blot. These observations were all prevented by AG490 inhibition of JAK2 activity. Our findings demonstrate that JAK2 regulates tIRI-induced GCA, oxidative DNA damage and activation of the ATM/Chk2/H2AX and ATR/Chk1 DDR pathways, but the cell made the apoptosis decision despite DDR efforts.

## 1. Introduction

Testicular ischemia-reperfusion injury (tIRI) is widely accepted as the underlying mechanism for testicular torsion and detorsion (TTD) [1]. The pathophysiology of testicular torsion, a urologic emergency, is an ischemic event due to the twisting of the spermatic cord obstructing blood flow to the testis [2]. Immediate surgical intervention is a must to detorse the twisted cord and reperfuse the testis in order to prevent testicular dysfunction. At the molecular level, reperfusion of ischemic tissues upon detorsion will result in the formation of toxic reactive oxygen species (ROS), which are inducers of oxidative stress (OS) due to their potent oxidizing and reducing abilities. Direct damaging effects of ROS extend from the plasma membrane to the nuclear DNA of the cell; thus ROS can jeopardize cellular integrity and function [3]. Since spermatogenesis is the main function of the testis, sperm DNA integrity is crucial for producing normal motile spermatozoa. Oxidative DNA damage and disruption of spermatogenesis in injured testis will lead to germ cell apoptosis (GCA) [1]. 

One of the main cellular pathways activated by the accumulation of intracellular ROS and OS is the JAK/STAT signaling pathway [4]. In the testis, the JAK/STAT pathway is involved in most of the male reproductive events, from sexual development to sperm maturation [5]. In addition, JAK/STAT was also reported to play a role in gametogenesis and spermatogonial stem cells generation and renewal, which are essential processes to ensure male fertility [6]. In spermatocytes, the JAK/STAT pathway is involved in the maintenance of meiotic chromosome structure and repair of meiotic double-strand breaks [5]. Hence, the JAK/STAT pathway is considered as a therapeutic target to treat spermatogenesis impairment [7]. During testicular OS, a major apoptosis-inducing factor is oxidative DNA damage. Intracellular ROS can directly damage DNA. Types of spermatozoa oxidative DNA damage identified include single-strand and double-strand DNA breaks (SSB and DSB), base modifications, abasic sites, and DNA-protein cross-links [8]. An increase in the 8-hydroxy guanine (8-OHdG) DNA adduct was reported to cause the formation of abasic sites that will destabilize the DNA structure and cause consequent DNA SSB and DSB. The most cytotoxic type of these damages is DNA DSBs. Accumulation of such DNA damages or the inability of the cell to repair them can result in the formation of oxidatively induced clustered DNA lesions (OCDLs) [9]. 

In the testis, unrepaired oxidative DNA damage is detected by the DNA damage response (DDR) pathways [10,11]. The DDR pathways consist of the ataxia–telangiectasia-mutated (ATM) and ataxia telangiectasia and Rad3-related (ATR) kinases, and their downstream checkpoint kinases Chk2 and Chk1, respectively. While the ATM pathway responds to DNA DSB, the ATR pathway responds to DNA SSB. To date, only two members of the STAT family, STAT3 and STAT5, were reported to modulate the ATM and the ATR pathways [12,13]. However, there is a lack of direct evidence linking JAK2 activation with the two DDR pathways.

To better understand the function of the ATM and ATR pathways and to establish their correlation with JAK2, a brief description of their activation events is introduced. The activation and signal transduction events within the DDR network are usually mediated by protein phosphorylation [14]. In brief, DNA DSBs are sensed by the Mre11-Rad50-Nbs1 (MRN) complex, which causes the autophosphorylation of ATM at Ser 1987 and its subsequent activation. Active ATM will phosphorylate its downstream substrates Chk2 and the histone H2AX at Thr 68 and Ser 139, respectively. On the other hand, DNA SSBs are sensed by the recruitment of the replication protein A heterodimers (RPA) at the site of damage [15,16]. DNA SSB-bound RPA will phosphorylate ATR at Ser 428, 435 and Thr 1989, which will, in turn, phosphorylate Chk1 at Ser 317 and Ser 345. Both Chk2 and Chk1 will act on a number of downstream substrates that will signal for cell cycle arrest and DNA repair. In case the damage cannot be removed or repaired, apoptosis is triggered. Activation of the ATM kinase and phosphorylation of its main substrate, H2AX, in spermatocytes of the normal adult human testis were detected by immunohistochemical staining in situ [17]. It has also been demonstrated that the mRNA and protein of ATR are highly expressed in human and mouse testes [18].

Noticeably, no previous study has investigated the presence of a direct link between JAK2 and the DDR pathways, particularly in an in vivo model of tIRI. To evaluate the role of JAK2 in tIRI-induced DNA damage and activation of the ATM/Chk2 and ATR/Chk1 DDR pathways AG490, a JAK2 specific inhibitor, was used in this study. We hypothesize that one or both of the DDR pathways might be activated during tIRI, which might be modulated by JAK2 signaling to induce GCA. Our results show that tIRI-induced oxidative DNA damage and GCA were prompted by activation of the JAK2/STAT1/STAT3 signaling pathway, which was found to upregulate the DDR signaling pathways ATM/Chk2 and ATR/Chk1 and prompted the apoptosis decision.

## 2. Results

### 2.1. The JAK2/STAT3/STAT1 Pathway Is Directly Involved in tIRI

**Figure 1:**
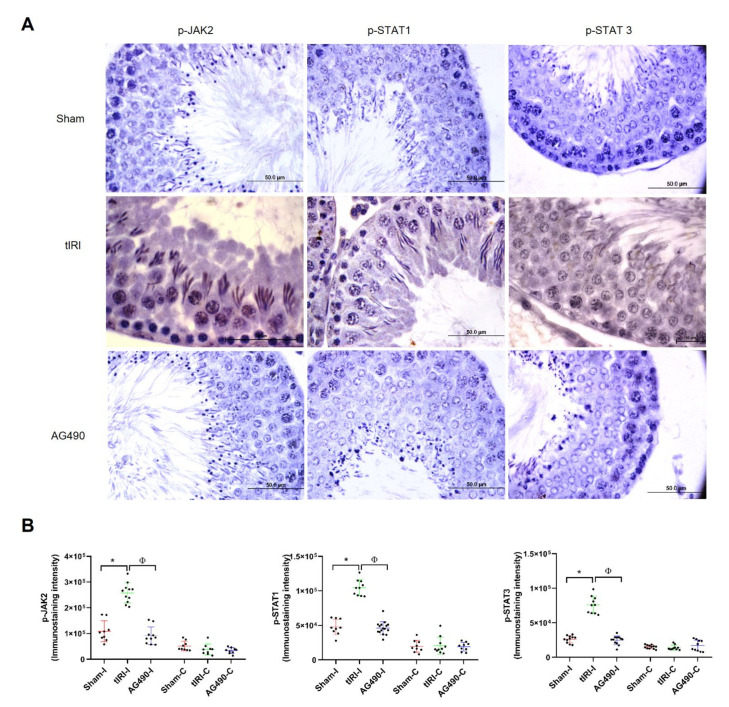
The JAK2/STAT1/STAT3 pathway is directly involved in tIRI. (**A**) Immunoexpression of p-JAK2/p-STAT1 and p-SAT3 in ipsilateral testicular tissue was detected and measured by IHC using the Cell Sens Dimension software. Prominent immunostaining and hence the expression of the phosphorylated forms of JAK2, STA1 and STA3 was detected in the tIRI seminiferous tubules (STs) in comparison to sham tissues. AG490 treatment (40 mg/kg) during ischemia and prior to reperfusion diminshed the immunoexpression of p-JAK2, p-STAT1 and p-STAT1 in the STs of rat testes. (**B**) Diagrams representing the immunoexpression of p-JAK2, p-STAT1 and p-STAT3. In comparison to the ipsilateral * sham and ^Φ^ AG490-treated groups, the ipsilateral tIRI testes showed higher p-JAK2 (* *p* < 0.0001, ^Φ^ *p* < 0.0001, respectively), p-STAT1 (* *p* < 0.0005, ^Φ^ *p* < 0.0001, respectively) and p-STAT3 (* *p* < 0.0001, ^Φ^ *p* < 0.0001, respectively) indicating activation of the JAK/STAT pathway during tIRI-induced oxidative stress. The contralateral testes in all groups showed no significant differences (*p* > 0.05). Data are presented as mean ± SD (*n* = 6/group). * tIRI compared to sham and ^Φ^ AG490 compared to tIRI. I = Ipsilateral and C = Contralateral.

To evaluate the inhibitory effect of AG490, we determined the activation of the JAK2/STAT1/STAT3 pathway by using their respective phospho-antibodies to quantify their phosphorylation levels in situ (Figure 1). In the ipsilateral testes, the results show that JAK2 phosphorylation increased by 2.4-fold in the tIRI group compared to sham group. The use of AG490 was able to decrease JAK2 phosphorylation to sham levels. Similar to JAK2, the phosphorylation levels of STAT1 and STAT3 were also considerably increased by 2.2-fold and 2.9-fold, respectively, in the tIRI group compared to sham. Their phosphorylation was abolished by AG490 treatment during ischemia prior to reperfusion. The contralateral testes in the three experimental groups showed low phosphorylation levels with no statistical significance. These data establish the activation and immediate role of JAK2/STAT1/STAT3 signaling pathway during tIRI.

### 2.2. JAK2 Inhibition Prevents tIRI-Induced Spermatogenic Arrest

**Figure 2:**
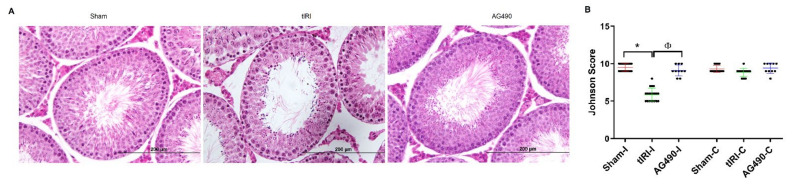
JAK2 inhibition prevents tIRI-induced spermatogenic arrest. (**A**) H&E stained sections of ipsilateral testes with 40x magnification representing the histological differences in the three experimental groups. Sham group sections had normal ST structure and spermatogenesis while the tIRI sections demonstrated disrupted tubular structure and disarrangement of germ cell layers. The AG490-treated tissue sections showed conserved morphology of the STs similar to sham group. (**B**) Graphs representing the histological analyses in ipsilateral and contralateral testes using the Johnsen score. The ipsilateral testis of tIRI group showed a lower Johnsen score when compared to * sham and ^Φ^ AG490-treated groups (Johnsen score: * *p* < 0.0001, ^Φ^ *p* < 0.0001, respectively). The contralateral testes in all groups showed no significant differences in the Johnsen score (*p* > 0.05). Data are presented as mean ± SD (*n* = 6/group). * tIRI compared to sham and ^Φ^ AG490 compared to tIRI. I = Ipsilateral and C = Contralateral.

The effect of tIRI on the testis morphological structure was assessed by H&E staining using the Johnsen’s score (Figure 2). The ipsilateral testis demonstrated a significant decrease in the Johnsen’s score during tIRI in comparison to sham and AG490-treated groups (5.85, 9.50 and 9.10, respectively). According to Johnsen’s scoring system, an average score of 5.85 describes the absence of spermatozoa and late spermatids with the presence of only few early spermatids. The contralateral STs in the sham, tIRI and AG490-treated groups displayed normal spermatogenesis with no significant differences in the Johnsen’s scores. The disrupted germ cell arrangement in the STs of ipsilateral testes and arrested spermatogenesis during tIRI are indicative of testicular dysfunction and germ cell death, which were all prevented by JAK2 inhibition.

### 2.3. JAK2 Inhibition Prevents Oxidative DNA Damage during tIRI

**Figure 3:**
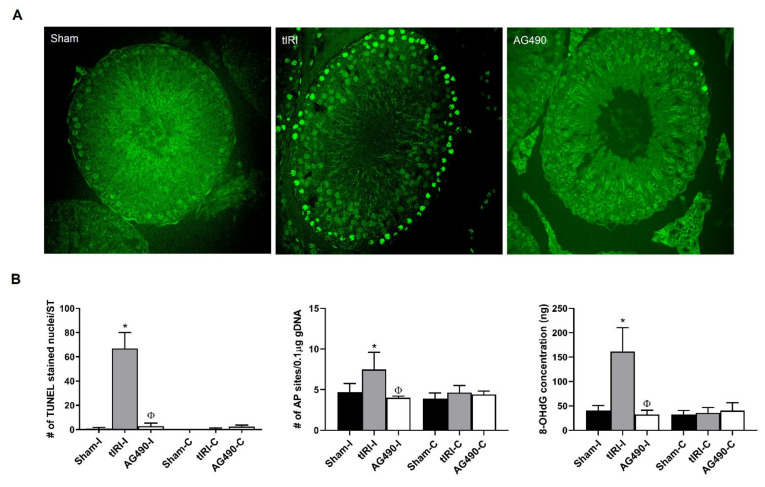
JAK2 inhibition prevents tIRI-induced oxidative DNA damage. (**A**) Representative images of TUNEL assay using fluorescently labeled 3′ dUTP of ipsilateral testes at 40x magnification. The tIRI histological sections had a significantly higher number of TUNEL-positive stained nuclei compared to sham. The AG490-treated tissue sections showed a diminished number of TUNEL-positive stained nuclei similar to sham. (**B**) A graph representation of oxidative DNA damage markers: DNA strand breaks (TUNEL), AP sites quantification and 8-OHdG concentration. Ipsilateral testes of tIRI group showed higher number of DNA strand breaks (* *p* < 0.0001, ^Φ^ *p* < 0.0001), number of AP sites (* *p* = 0.0005, ^Φ^ *p* < 0.0001) and 8-OHdG concentration (* *p* = 0.0005, ^Φ^ *p* < 0.0001) compared to sham and AG490-treated groups, respectively. The contralateral testes in all groups showed no significant difference (*p* > 0.05). Data are presented as mean ± SD (*n* = 6/group). * tIRI compared to sham and ^Φ^ AG490 compared to tIRI. I = Ipsilateral and C = Contralateral.

In order to assess the degree and nature of tIRI-induced oxidative DNA damage, the following markers were detected: DNA SSBs and DSBs, AP sites and 8-OHdG. Both DNA SSBs and DSBs were detected by the in situ TUNEL assay, while AP sites and 8OHdG were quantified using colorimetric assays (Figure 3). The tIRI-subjected ipsilateral STs displayed a striking increase in the number of TUNEL stained nuclei/ST (80-fold increase) in comparison to sham and AG490-treated STs. This was paralleled with a considerable increase in the concentrations of both AP sites (1.6-fold) and 8OHdG (4-fold) in the tIRI group compared to sham and AG490 groups. The contralateral testes in the three experimental groups all had baseline levels of DNA damage with no significant differences between them. The aberrant accumulation of oxidative DNA lesions during tIRI explains the high impact of tIRI-induced ROS on the process of spermatogenesis and arresting the development of mature germ cells. However, the inhibition of JAK2 activity abolished the rate of DNA damage, maintained genome stability and protected against germ cell death, thus supporting the involvement of JAK2 in germ cell death during tIRI.

### 2.4. JAK2/STAT1/STAT3 Activation Promotes Apoptosis during tIRI

**Figure 4:**
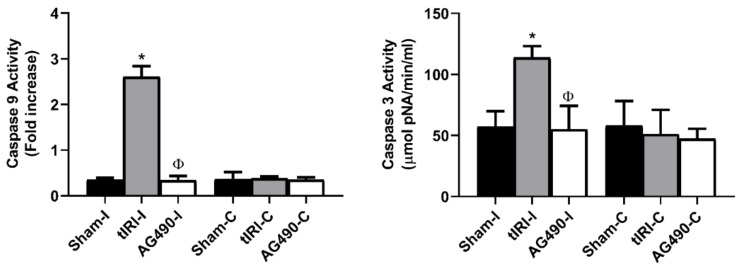
JAK2 activation promotes apoptosis during tIRI. Induction of the caspase cascade in response to JAK2 activation was demonstrated by the increased activities of the initiator caspase 9 and the executioner caspase 3 during tIRI compared to sham (* *p* < 0.0001). Treatment with AG490 (40 mg/kg) prior to reperfusion suppressed the activation of caspases 9 and 3 (^Φ^ *p* < 0.0001). The contralateral testes in all groups showed no significant difference (*p* > 0.05). Data are presented as mean ± SD (*n* = 6/group). * tIRI compared to sham and ^Φ^ AG490 compared to tIRI. I = Ipsilateral and C = Contralateral.

Caspases 9 and 3 are key players during the demolition phase of apoptosis. To show whether they are influenced by JAK2 activation, their activities were measured by colorimetric assays in the absence and presence of AG490 (Figure 4). The enzyme activity of the initiator caspase 9 was elevated by 7.4-fold compared to sham and AG490-treated groups. Similarly, the activity of the major executioner caspase 3 was also considerably elevated by 2-fold in the tIRI group compared to sham and AG490-treated groups. The contralateral testes of the three animal groups showed baseline activity of caspases 9 and 3. These data clearly indicate that the apoptotic activity of both caspases are regulated by JAK2 signaling since their activities were inhibited by AG490 and GCA was prevented.

### 2.5. PARP Activity Is Regulated by JAK2

**Figure 5:**
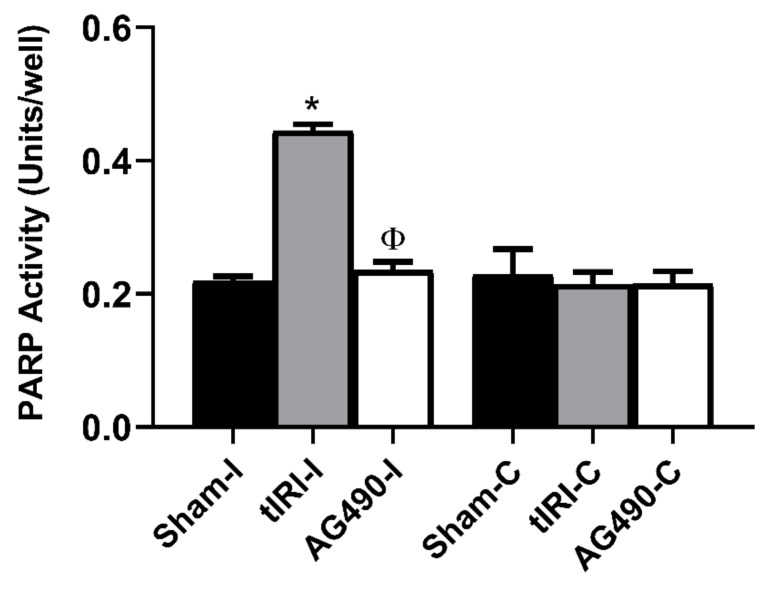
PARP activity is regulated by JAK2. PARP activity was measured colorimetrically and expressed as units/well. The ipsilateral testes of tIRI had significantly higher activity of PARP compared to sham and AG490-treated groups (* *p* < 0.0001, ^Φ^ *p* < 0.0001). The contralateral testes in all groups showed no significant difference in PARP activity (*p* > 0.05). Data are presented as mean ± SD (*n* = 6/group). * tIRI compared to sham and ^Φ^ AG490 compared to tIRI. I = Ipsilateral and C = Contralateral.

PARP is activated by oxidative stress, acts as a sensor of DNA damage and recruits DNA repair components. The decision can either be to repair DNA damage or to degrade DNA and trigger cell death. The enzymatic activity of PARP was increased in tIRI-subjected testes by 2-fold compared to sham and AG490-treated (Figure 5). Inhibition of JAK2 activity by AG490 resulted in the loss of PARP activity and confirmed its role during testicular stress and GCA.

### 2.6. Modulation of the DDR Pathways by JAK2 Signaling

**Figure 6:**
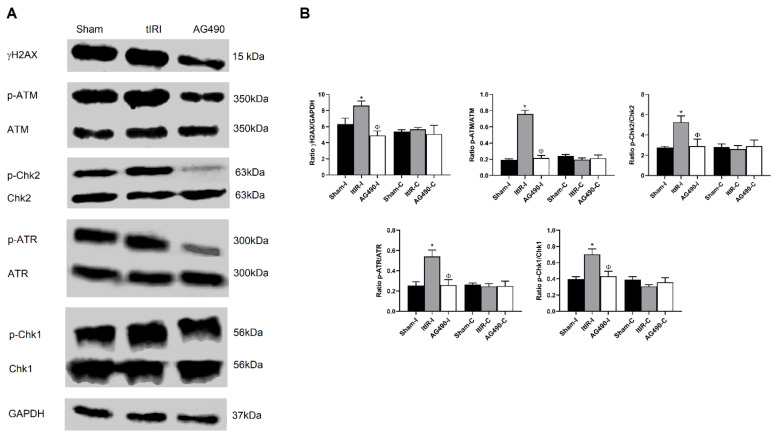
Modulation of the DDR pathways by JAK2 signaling. (**A**) Representative Western blots showing changes in the phosphorylation levels of γH2AX, ATM, Chk2, ATR and Chk1. (**B**) Testes subjected to tIRI showed a marked elevation in the expression of γH2AX, p-ATM, p-Chk2, p-ATR and p-Chk1 compared to sham (*p* = 0.0470, *p* = 0.0002, *p* < 0.0001, *p* < 0.0001, respectively), while treatment with AG490 (40 mg/kg) resulted in a considerable decrease in their expression compared to tIRI levels (*p* = 0.0051, *p* < 0.0001, *p* = 0.0002, *p* < 0.0001, respectively). Contralateral testes in all groups showed similar protein expression with no significant differences (*p* < 0.05), which are presented in Supplementary Figure S1. Data are presented as mean ± SD (*n* = 3/group). * tIRI compared to sham, ^Φ^ AG490 compared to tIRI. I = Ipsilateral and C = Contralateral.

The ATM/ATR DDR pathways are two of the most predominant signaling pathways that regulate cell-cycle arrest, DNA replication, DNA repair and apoptosis. Previous studies have reported the involvement of some STAT members in the DDR pathways. Consequently, in the current study we investigated the role of JAK2 in the modulation of the γH2AX/ATM/ATR pathways during tIRI by Western blot (Figure 6). In the tIRI-subjected testis, the expression levels of γH2AX, p-ATM, p-Chk2, p-ATR and p-Chk1 were markedly increased by 1.4-, 4.0-, 1.9-, 2.2- and 1.8- folds, respectively. However, AG490 treatment prior to reperfusion considerably decreased the expression levels of these phospho-proteins by 1.75-, 3.6-, 1.8-, 2.1- and 1.6- folds, respectively. These results validate that the activation and signaling of JAK2 and its downstream STATs 1 and 3 are necessary for the repair of tIRI-induced oxidative DNA damage to some extent via the activation of the γH2AX/ATM/Chk2 and ATR/Chk1 pathways.

## 3. Discussion

The most detrimental effect of IRI-induced-oxidative stress is inflicted on the DNA of testicular germ cells and their subsequent death by apoptosis that might lead to testicular atrophy and impaired spermatogenesis [19,20]. Here we tested the hypothesis that JAK2 signaling could promote oxidative DNA damage and GCA via modulation of the DDR pathways during tIRI. Our findings supported the direct regulation of JAK2 of oxidative DNA damage and activation of the two DDR pathways induced by tIRI. This was verified by the AG490 treatment during ischemia and prior to reperfusion, which inhibited JAK2 activation and maintained genome stability and survival of germ cells.

Excessive generation of ROS is a key trigger of JAK/STAT signaling [4]. Increased ROS levels activate the JAK2/NFκB pathway in the testis, which triggers apoptosis and results in dysfunctional testicles, while JAK2 inhibition reduces oxidative damage and cell apoptosis in the testis [21,22]. In vitro, JAK/STAT phosphorylation was increased in high glucose, and quercetin treated Sertoli cells, implicating JAK’s role in diabetes-induced testicular damage [23]. Upon activation by JAK2, the roles of STAT1 and STAT3 in cell proliferation and apoptosis is still controversial, with some studies associating the two STATs with either function depending on the type of tissue and the nature of the injury [24]. But, the two STATs share the same modus operandi where they bind their respective DNA elements in the promoter region of apoptosis-related genes and modulate their expression. Interestingly, it was reported that STAT1 but not STAT3 forms protein-protein interactions with p53. The STAT1/p53 complex enhances the activity of pro-apoptotic genes by the binding of p53, but not STAT1, to the promoter region of these genes [25]. The STAT1^−/−^ cells were shown to constitutively express caspases 1, 2 and 3, and some pro-apoptotic genes like Fas, FasL, p21 and p53 and hence are resistant to TNF-induced apoptosis [26]. STAT1 is cleaved by caspase-3 and the pro-apoptotic C-terminal of STAT-1 is released to propagate the apoptosis cascade during IRI [27]. The STAT1-activated p53 can also activate caspase-9 during apoptosis, which in turn activates caspase-3 and downstream effectors. 

However, caspase 3 can also activate more caspase 9 via feedback amplification at later stages of apoptosis [28]. As an anti apoptotic STAT, STAT3 facilitates cell survival by activation of several bcl-2 family members, the pim kinases, and the IAP protein survivin. STAT3 was shown to promote cell survival by associating with c-jun to suppress transcription of the pro-apoptotic cytokine Fas [29]. In addition, inhibition of STAT3 by AG490 led to the activation of ERK and promoted cell survival during oxidative stress [30]. If STAT3 is expected to have an antiapoptotic role during OS-induced apoptosis, then it is paradoxical that the phosphorylation of both STAT1 and STAT3 was reduced by AG490 treatment in our current in vivo tIRI model. This could suggest that the cell’s response to IRI is based on the balance between the pro-apoptotic STAT1 and the anti-apoptotic STAT3. These studies are consistent with our findings where AG490-inhibited JAK2/STAT1/STAT3, attenuated caspases activation and prevented GCA. This confirms the crucial role of JAK2 signaling in triggering apoptosis in oxidative stress-challenged cells.

The role of JAK2/STAT1/STAT3 pathway was investigated in different organ IRI models. In these models, AG490-mediated inhibition of the JAK/STAT1/STAT3 pathway resulted in reduced oxidative damage, inhibition of apoptosis and normalized tissue function, which was partly attributed to the expression of oxidative stress-responsive molecules involved in DNA damage, DNA repair and apoptosis. We have previously reported on the involvement of JAK phosphorylation as part of the SAFE pathway during tIRI [31]. In hypoxic rats, the activation of the JAK2/STAT6 pathway resulted in testicular damage and oligozoospermia [32]. The phosphorylation of JAK2 and its downstream molecules STAT1 and STAT3 was significantly heightened during hepatic IRI [33]. 

In cerebral IRI, administration of a ROS scavenger prior to ischemia suppressed STAT3 activation indicating the link between ROS production and STAT3 phosphorylation [34]. Another earlier study reported that deletion of STAT1 gene increased the resistance of the brain tissue against the ischemic injury [35]. The ability of AG490 to inhibit JAK2 activity and suppress the activation of STATs 1 and 3 has rescued the kidneys from renal failure during IRI [36]. Similarly, blockage of the JAK/STAT signaling pathway using the AG490 prevented intestinal injury following IRI as demonstrated by reduced Chiu’s score and intestinal mucosal lactic acid level [37]. The role of the JAK/STAT pathway in organ IRI is consistent with our current data, since the use of AG490 blocked JAK/STAT activation and counteracted tIRI-induced OS.

The excessive production of ROS and creating a microenvironment of OS is a key mediator of sperm dysfunction due to the lack of cytoplasm and antioxidant enzymes in spermatozoa [1]. Upon oxidation, the polyunsaturated fatty acids in the sperms’ plasma membranes will generate highly reactive aldehydes that will react with nucleic acids and amino acids, forming stable DNA and proteins adducts that will eventually decrease membrane fluidity [1]. In normal human cells, about 1% of DNA SSB are transformed to roughly 50 DNA DSB/cell/cell cycle [38]. Such random stimulation of DNA DSBs is usually very fast, within seconds or a few minutes. In addition, physiological levels of ROS were shown to significantly increase the formation of OCDLs that would consequently result in triggering replication-independent DNA DSB [9]. 

These reports confirm the genotoxic effects of DNA DSB during oxidative stress conditions. Previously, we have demonstrated the formation of γH2AX foci in testicular tissue subjected to tIRI by immunofluorescence staining [39]. Here, the 1.4-fold increase in the protein expression of γH2AX in parallel to extensive tIRI-induced DNA damage suggests the formation of a γH2AX foci in preparation for DNA damage response and subsequent DNA DSB repair. However, the vast accumulation of DNA strand breaks (by 80-folds) after 4 h of reperfusion could have disrupted the phosphorylation and dephosphorylation cycles of the histone H2AX, which could prevent its recognition by the DNA damage repair proteins. This could also result in defective chromatin remodeling during the rapid cell divisions of spermiogenesis and increase the DNA’s risk of ROS attacks. But germ cells might not always be able to repair nucleotide modifications and DNA strand breaks due to their cellular nature. 

During spermiogenesis, haploid spermatids are suggested to rely mainly on the error-prone non-homologous end joining (NHEJ) pathway to repair their DNA strand breaks due to their inability to undergo homologous recombination (HR) [40]. Spermiogenesis is characterized by rapid cell divisions, diminished antioxidant protection and partial inactivation of some DNA repair enzymes involved in NHEJ, such a combination of events could subject spermatids to genomic instability and negatively affect their function even under normal oxygen tension [41]. Therefore, in the absence of an efficient NHEJ repair system, dysregulated activation of H2AX and increased oxygen tension during tIRI, germ cells are forced to trigger apoptosis. Oxidative DNA damage is a plausible cause for defective spermatozoa. [42]. It was reported that 20–88% of subfertile men had elevated levels of ROS in their semen [43]. In asthenospermia, subfertility and DNA damage were all attributed to increased concentrations of ROS [44]. Here, we showed that tIRI-induced DNA lesions and low Johnsen scores were prevented by inhibiting JAK activation, implicating its crucial role in inducing oxidative DNA damages and spermatogenic arrest in tIRI.

Like the JAK/STAT pathway, pathological intracellular ROS levels will also trigger the activation of the ATM pathway due to the accumulation of DNA strand breaks [45]. The first link between JAK/STAT signaling and the ATM pathway was established in an in vivo model of IRI [46]. It was demonstrated that H2AX phosphorylation took place during reperfusion but not ischemia and had a similar phosphorylation rate to that of STATs 1 and 3, suggesting a parallel activation of the ATM and JAK/STAT pathways. Since STAT1 was shown to activate p53, it could be suggested that STAT1 might also regulate the ATM pathway via the ATM/p53 pathway [47]. Upon DNA damage and DDR activation, p53 might upregulate the expression of the pro-apoptotic genes Bax and PUMA to trigger apoptosis. In parallel, p53 could stimulate mitochondrial apoptosis leading to cytochrome c release and activation of the caspase cascade [48]. 

Interestingly, PARP is required for proper p53 activation and stabilization [49]. It is also required for ATR function during replication stress as demonstrated by their protein-protein interactions [50]. However, PARP can be described as a double-edged sword due to its ability to regulate apoptosis and cell proliferation. PARP is known as a sensor of DNA breaks, and its activity is essential for the repair of single-strand DNA breaks via the base excision repair pathway [51], but its ability to enhance the expression of iNOS and NFkB implicates its indirect involvement in the pathogenesis of IRI [52]. Furthermore, PARP activity was found to be regulated by the IL6/JAK2/STAT3 pathway via NFkB transactivation [53]. Few in vitro studies were conducted to investigate the relation between JAK/STAT and ATM/ATR DDR pathways. 

UVA induction of Ser727 phosphorylation of STAT1 and STAT3 but not basal protein expression was reduced in ATM-deficient cells compared to control [54]. A reduction in ATM/Chk2 and ATR/Chk1 signaling was shown in STAT3 deficient cells by altering the phosphorylation of H2AX Chk1 proteins [12]. In anogenital cancers, STAT5 downstream targets were recognized as members of the ATM pathway, consequently linking the JAK/STAT pathway with DNA damage [13]. Furthermore, inhibition of JAK/STAT signaling eliminated the ATM pathway-mediated DNA repair activity in hepatocytes [55]. The gonads of A-T patients and *ATM-deficient* mice were found to lack germ cells, while spermatocytes from *ATM^−/−^* mice suffered from meiotic arrest in early prophase with increased chromosome fragmentation during the prophase stage [56,57]. Thus, cellular response to DNA damage via the DDR pathways is essential for maintaining genomic integrity.

The involvement of the ATM/ATR and their related proteins were reported to have marked roles in some organ IRI models. In a focal cerebral IRI model (i.e., experimental stroke), there was a prominent formation of oxidative DNA lesions like 8OHdG, DNA SSB and DSB, fragmented 3′-OH DNA ends, and apoptosis [58]. In line with this inference, increased concentrations of superoxide anions was reported in the cerebella of *ATM^−/−^* mice [59]. During renal IRI, the ATM/ATR-Chk2/1 pathways were activated in response to DNA damage leading to G2 arrest [60]. In line with this inference, our observation of the simultaneous activation of the JAK/STAT and ATM/ATR pathways during tIRI and their paralleled inhibition by AG-490 treatment suggests the presence of cross-talk between the two pathways during tIRI to decide on the cells’ fate to live or die. However, overwhelming ROS-induced DNA damage was beyond repair; thus, the cell death decision was opted.

In summary, the current study demonstrates the involvement of JAk2 signaling in tIRI-induced GCA and its inhibition provides protection against oxidative stress and oxidative DNA damage-induced GCA. Our findings also revealed for the first time a direct association between JAK2 activation and the two DDR pathways ATM/Chk2 and ATR/Chk1, which were found to be regulated by JAK2 signaling. This suggests that the actions of the pro-apoptotic STAT1 and anti-apoptotic STAT3 are in play during tIRI due to the parallel activation of the caspase death pathway and the ATM/ATR pathways. Future investigations could focus on the mechanism of cross-talk between JAK2 and the DDR pathways by examining more components from the two pathways using proteomics.

## 4. Materials and Methods

### 4.1. AG490 and Primary Antibodies

For JAK2 inhibition, Tyrphostin AG490 (#3434) was purchased from Sigma-Aldrich Co. (St. Louis, MO, USA). Antibodies against p-JAK2 (ab195055) and ATM (2CI) were purchased from Abcam (Cambridge, UK) and Santa Cruz Biotechnology (Dallas, TX, USA), respectively. Antibodies against Chk1(LS-C352010), p-Chek1 (LS-C358948), Chk2 (LS-C358943), p-Chk2 (LS-C416418), p-ATM (LS-C353715) were all purchased from Lifespan BioSciences (Seattle, WA, USA). Antibodies against p-STAT1 (#8826) and p-STAT3 (#9145), γH2AX (#9718), ATR (#2790), p-ATR (#2853) and GAPDH (#2118) were purchased from Cell Signaling Technology (Danvers, MA, USA). The secondary antibodies for Western blot: peroxidase-conjugated AffiniPure Goat anti-Rabbit (#133499) and anti-Mouse (#133599) were purchased from Jackson ImmunoResearch (West Grove, PA, USA). For anesthesia, Ketamine and Xylazine were purchased from Hikma Pharmaceuticals (Amman, Jordan) and Bayer GMP (Bergkamen, Germany), respectively.

### 4.2. Testicular Ischemia Reperfusion Injury (tIRI) Model and AG490 Treatment

Male Sprague-Dawley (SD) rats (Charles River, Waltham, MA, USA) aging 8 weeks old and weighing 250 to 300 g, were used and raised according to the recommendations of Kuwait University guidelines of experimental animals. Rats were supplied with food and water *ad libitum* and maintained on a 12 h’ light/12 h’ dark cycle. The surgical procedure was approved by the ethics committee on animal research at Kuwait University.

The rats (*n* = 36) were randomly and equally divided into three groups: sham, tIRI only and tIRI + AG490 [39]. All rats were subjected to anesthesia using a mixture of ketamine (50 mg/kg) and xylazine (2 mg/kg). The surgical site was shaved and disinfected using 70% ethanol and Betadine. Sham rats underwent a regular ilioinguinal incision at the left side and the left ipsilateral testis was exposed for 60 min before returning it into the scrotal sac. The incision was then closed with surgical clips and sham rats were sacrificed after 4 h [1]. The tIRI only rats experienced a unilateral ischemic injury using a microvascular surgical clamp with 700 g of pressure that clipped the left testicular artery and obstructed the blood supply to the ipsilateral testis. After 30 min of ischemic injury induction, injured rats were injected intraperitoneally (i.p.) with DMSO (drug vehicle). The clamp was removed after 1 h of ischemia to allow testicular reperfusion for 4 h before animal sacrifice. The tIRI + AG490 rats were treated similarly to the tIRI group except for the rats were injected i.p., with the JAK kinase inhibitor Tyrphostin AG490 (40 mg/kg) instead of DMSO [33]. The right contralateral testes were used as an internal positive control in all investigations.

### 4.3. Histological Analyses

Harvested testes (ipsilateral and contralateral) were immediately fixed in Bouin’s solution (50% saturated picric acid, 35% distilled water, 10% formalin and 5% acetic acid) and processed before embedding in paraffin blocks. Testicular tissue sections (4 µm) were stained with hematoxylin and eosin (H&E) and examined under the Zeiss LSM 700 light microscope (Carl Zeiss Microscopy Ltd. Jena, Germany) using 10×, 20× and 40× magnifications. A total of 50 seminiferous tubules (STs) were used for evaluating spermatogenesis using the Johnsen’s score [61]. The 10 points scoring system quantifies spermatogenesis. A score of 10 represents complete spermatogenesis, whereas a score of 1 means lack of germ cells in the STs. The examiner was blinded to the different groups analyzed.

Immunohistochemistry (IHC) staining was performed using the Histostain plus Broad Spectrum kit (Life Technologies, Carlsbad, CA, USA) following the company’s instructions. Testicular tissue sections (4mm) were de-waxed, rehydrated and retrieved through immersion in citrate buffer (18% citric acid monohydrate and 82% sodium citrate dehydrate to yield 0.01 M citrate buffer, pH = 6). The slides were then microwaved and washed with PBS. Afterward, the slides were treated with 3% H_2_O_2_, washed, and blocked using a blocking solution. The processed slides were then incubated overnight at 4 °C with their respective primary antibodies (p-JAK2, p-STAT1, p-STAT3 at 1:100 dilution). Unbound antibodies were washed off and incubated with broad spectrum secondary antibody followed by HRP-streptavidin treatment. Using the DAB impact kit (Vector Laboratories, Burlingame, CA, USA), DAB was added to the slides until the desired brown color was observed under a light microscope. The slides were then stained with hematoxylin, washed, and dehydrated. Immunostaining intensity was quantified [intensity = area (number of pixels^2^)] using the Cell Sens Dimension Software (Olympus DP 71 camera, Olympus, Tokyo, Japan). Six slides (two slides/rat) were used to evaluate each experimental group. Additionally, the color intensities from 10 to 15 STs at 10× magnification were randomly scored and averaged from each section within each group. Image analysis and immunostaining scoring were performed in a blinded manner.

### 4.4. Protein Extraction and Western Blot

The radio-immunoprecipitation assay (RIPA) lysis buffer (Santa Cruz Biotechnology, Dallas, TX, USA) mixed with a cocktail of protease inhibitors was used to homogenize frozen testicular tissue. Total protein concentration was measured at 595 nm using the Ultrospec 2100 pro UV visible spectrophotometer (Biochrom, Holiston, MA, USA). Protein samples were stored at −80 °C. Depending on the nature of detected proteins (phosphorylated and un-phosphorylated), 50–200 µg of total cell extracts were electrophoresed on SDS-polyacrylamide gels (10–12%), transferred to PVDF membranes and blocked in 5% non-fat dry milk in PBS-Tween. Membranes were then incubated overnight at 4 °C with specific primary antibody (1:1000 dilution: Chk1, Chk2, p-Chk2, ATR, p-ATR, GAPDH; 1:500 dilution: p-Chk1, p-ATM, γH2AX; 1:100 dilution: ATM). Washed PVDF membranes were incubated with peroxidase-conjugated secondary antibodies (1:4000 dilution) with gentle shaking. The protein-antibody signals were amplified using the ECL chemiluminescence kit (GE Healthcare, Amersham, Buckinghamshire, UK), visualized using the ChemiDoc imaging system and band intensities were quantified with the Image Lab software (BIO-RAD, Hercules, CA, USA).

### 4.5. Caspase Activity

Activation of the apoptosis cascade was determined by measuring the activities of the initiator caspase 9 and the executioner caspase 3. A standardized protein concentration was determined individually for each assay. The caspase-9 colorimetric assay kit (BioVision, Milpitas, CA, USA) and the caspase 3 assay Kit (Sigma-Aldrich, St. Louis, MO, USA) were used to measure the enzyme activity of caspases 9 and 3, respectively. All assays were performed following the manufacturer’s protocols.

### 4.6. Genomic DNA (gDNA) Extraction and Oxidative DNA Damage Assays

Total genomic DNA (gDNA) was extracted from frozen testicular tissues using the DNeasy Blood & Tissue Kit (Qiagen, Hilden, Germany). The purity of isolated gDNA was determined using the EPOCH Nanodrop spectrophotometer and the Gen software (Bio Tek Instruments, Winooski, VT, USA). The extraction of gDNA was performed following the manufacturer’s protocol. Purified gDNA was used to perform the following oxidative DNA damage assays.

The concentration of 8-OHdG nucleotide (ng) was measured in 300ng of gDNA/sample using the Epiquik^TM^ 8-OHdG DNA Damage Quantification Direct Kit (Epigentek, Farmingdale, NY, USA). In this assay, the 8-OHdG nucleotide was detected by capture and detection antibodies and quantified colorimetrically at OD_450nm_ using a microplate reader. The assay was performed following the manufacturer’s protocol.

The DNA damage quantification colorimetric kit (Biovision, Milptas, CA, USA) was used to measure the level of Apurinic/Apyrimidinic (AP) sites in 0.1 μg gDNA sample. The assay was performed following the manufacturer’s protocol.

DNA strand breaks were detected in testicular tissue sections (4 μm) by the TUNEL assay using the In Situ Cell Death Detection Kit, POD (Penzburg, Germany). TUNEL-stained slides were examined with the Zeiss LSM 700 confocal microscope using the ZEN Black and ZEN Blue software (Carl Zeiss Microscopy Ltd. Jena, Germany). A total of 50 STs per group were analyzed to quantify fluorescently stained nuclei. The assay was performed following the manufacturer’s protocol. The examiner was blinded to the different groups analyzed.

The ribosylation activity of PARP in response to DNA damage within testicular tissue was determined using the PARP/Apoptosis universal colorimetric assay kit (R&D Systems, Minneapolis, MN, USA). The assay was performed following the manufacturer’s protocol.

### 4.7. Statistical Analysis

For statistical analysis of raw data, GraphPad Prism v8.0 (GraphPad Software Inc., San Diego, CA, USA) was used. The one-way analysis of variance (ANOVA) test followed by Holm–Sidak’s multiple comparisons were used to compare the means for all experimental groups. Data were represented as mean ± standard deviation (SD) and the significant difference was fixed at *p* < 0.05.

## Data Availability

The data that support the findings of this study are available from the corresponding author upon reasonable request.

## Supplementary Materials

Supplementary Materials are available online at https://www.mdpi.com/article/10.3390/ijms222413390/s1.
