# Supplementary figures and images for: JAK Inhibition Prevents DNA Damage and Apoptosis in Testicular Ischemia-Reperfusion Injury via Modulation of the ATM/ATR/Chk Pathway
Source: Int J Mol Sci. 2021 Dec 13;22(24):13390. doi: 10.3390/ijms222413390 (PMC8706947; doi:10.3390/ijms222413390)

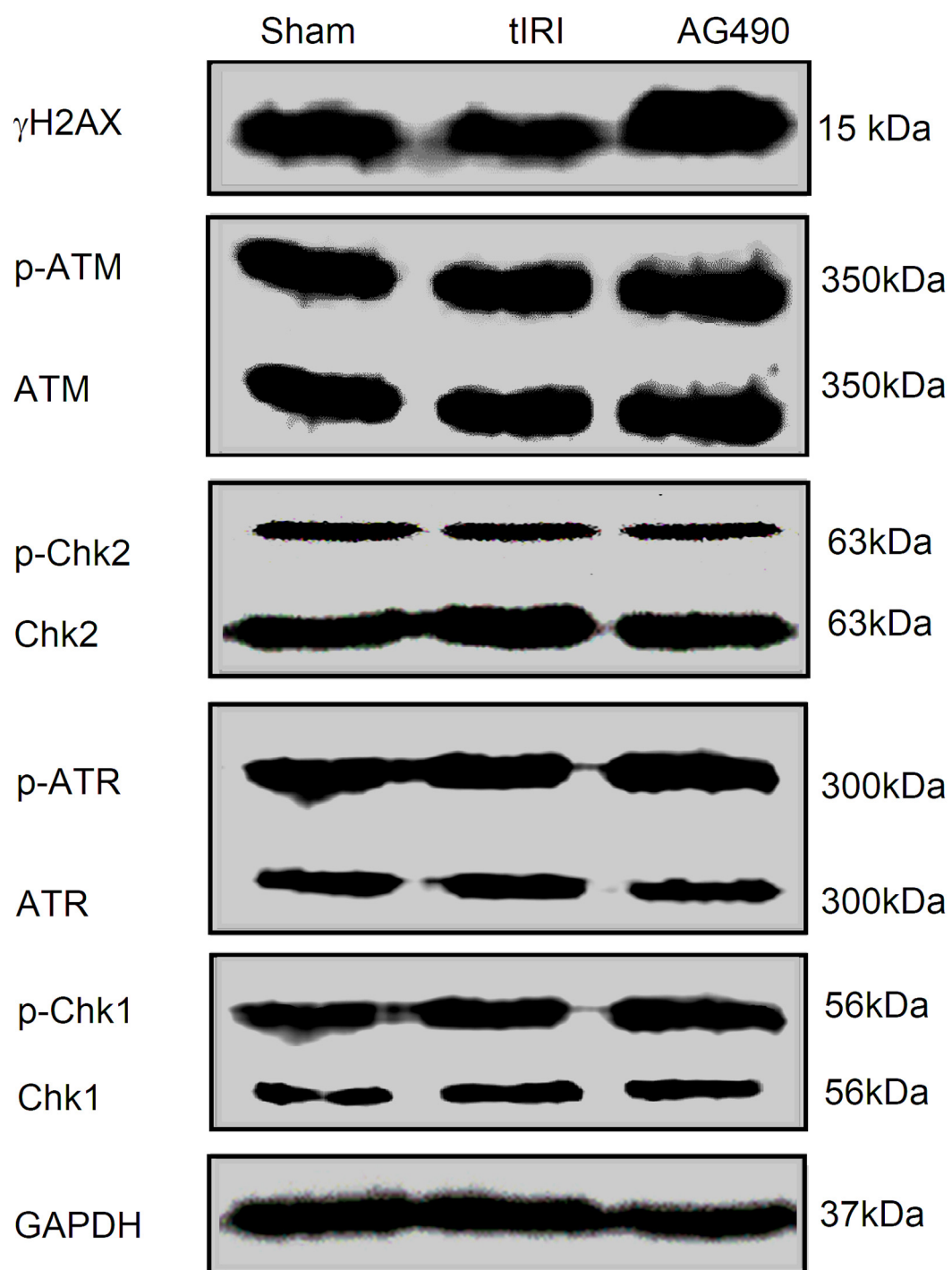

Supplement: Supplementary file 1 [file ijms-22-13390-s001.zip › ijms-1494785-supplementary.pdf]
